# Editorial: Causes and Consequences of Intrauterine Growth Restriction

**DOI:** 10.3389/fendo.2020.00205

**Published:** 2020-04-15

**Authors:** Ivo Bendix, Suzanne L. Miller, Elke Winterhager

**Affiliations:** ^1^Department of Pediatrics I, Neonatology and Experimental Perinatal Neurosciences, University Hospital Essen, Essen, Germany; ^2^Department of Obstetrics and Gynaecology, The Ritchie Centre, Hudson Institute of Medical Research, Monash University, Clayton, VIC, Australia; ^3^Imaging Center Essen, University Duisburg-Essen, Essen, Germany

**Keywords:** intrauterine growth restriction, fetal growth restriction, IUGR, FGR, pathology, placenta, brain injury

The origins of health and disease can be programmed during prenatal development caused by an unfavorable intrauterine environment, known as “fetal programming.” Intrauterine or fetal growth restriction (IUGR/FGR) describes a pathological condition in which the fetus fails to grow to its biological potential, primarily because of poor placental function. In turn, FGR is known to lead to short- and long-term consequences, like cardiovascular, renal, immunological and neurological disease that greatly impact on individuals and society. Adequate diagnostic approaches and therapeutic interventions are lacking. Thus, dissecting cellular and molecular mechanisms causing placental dysfunction, suboptimal fetal/offspring organ development, and disturbed genetic/epigenetic programming in the offspring are great challenges all required so that interventions can be explored. Defining individual causes for FGR will enable a better diagnosis and raise possible translational candidates for intervention to improve the health of the FGR offspring.

The aim of the present collection “Causes and Consequences of Intrauterine Growth Restriction (IUGR)” was to bring together the many causes for fetal growth restriction, and to describe the consequences of predisposition toward various diseases in later life. The 21 contributions, both reviews and original research papers, include basic science and clinical studies to highlight the placental origins of growth restriction, difficulties associated with *in utero* diagnosis, possible mechanisms initiating an adverse maternal environment, together with the consequences of fetal programming for neonatal and life-long health, plus discussion on potential interventions.

In putting together this collection of studies we became even more aware how diverse this problem is, and that we remain far away from identifying molecular mechanisms in common, or to ascertain a simple concept about the origin of health and disease. After a short discussion with Salavati et al., one of the contributing authors, the guest editors of this collection would like to shift the term intrauterine growth retardation/restriction (IUGR), toward the term fetal growth restriction (FGR). As investigators in the field, we agreed that the term FGR more appropriately addresses that it is the individual rather than the environment that is at risk. Furthermore, we would like to encourage a shift away from using the terms small for gestational age (SGA) and FGR interchangeably—in past years both terms have described an estimated fetal weight/ birth weight <10th centile, but SGA should not be used as a synonym for FGR. Most importantly, the difference between SGA and FGR refers to the fact that FGR fetuses are pathologically growth-restricted and demonstrate a reduction in growth trajectory as placental function fails. SGA describes a fetus that is small, but with a normal growth trajectory and normal umbilical artery Doppler velocimetry that indicate the SGA fetus is growing constitutively small along its genetically determined size. In contrast, FGR fetuses do not reach their genetically determined potential size due a pathological interruption to normal growth, most often resulting from placental insufficiency. Thus, SGA defines a statistical deviation only of size but not the pathological condition. Moreover, it would be worthwhile to address a subdivision of early-onset FGR (<32 weeks), and late-onset FGR (≥32 weeks) in future investigations because the two different time intervals are probably due to different causes of placental dysfunction and certainly result in varied consequences for the growth restricted infant.

Besides genetic defects or intrauterine infections, FGR is primarily attributed to a dysfunctional placenta. Lessons from mutant mice demonstrate that defective placental structures are clearly related to FGR. The review of Woods et al. summarizes studies of common types of placental defects in established mouse mutants, which help to instruct us in gaining a better understanding of placental gene expression pattern with an impact on failures in human placentation. In addition, gene expression responsible for the development of the different placental structures is often modulated by epigenetic mechanisms.

Identifying impairment of placental development is another challenge; Salavati et al. show that screening of defective human placental development by morphometry could serve to identify FGR at early stages for a better clinical management.

Placental dysfunction and reduced nutrient transfer to the fetus is a primary mediator of reduced fetal growth, however the placenta may be able to compensate and thus sustain the health of the developing fetus. Using mouse mutants defective in IGF2, Hayward et al. demonstrated that the placenta is able to compensate for the calcium transport by a decrease in calcium binding proteins, the parathyroid hormone-related protein and an increase in serum/glucocorticoid-regulated kinase. Another compensating mechanism is described by Schmidt et al. using a FGR rat model fed with low protein diet during pregnancy, which revealed that offspring showed significantly reduced levels of local corticosterone, probably a mechanism to sustain brain development. Endogenous compensation mechanisms are rarely investigated within FGR research but could help to identify novel treatment options in future. Placental infection during pregnancy is also a main cause FGR, and the review of Seitz et al. describes the molecular mechanisms of Plasmodium falciparum infection—still an important issue and reason for FGR in tropical/ subtropical regions—via a defined surface antigen. Deciphering such infection strategies might give rise to intelligent treatment options.

The pregnancy disease preeclampsia is a primary complication associated with FGR, and with an increase in the anti-angiogenic soluble fms-like tyrosine kinase 1 (sFlt-1) of the placenta. Vogtmann et al. presents an hs-Flt-1 overexpressing mouse model which evidenced that sFlt-1 is responsible for the development of FGR because of a reduced placental efficiency with changes in the maternal/fetal vasculature.

Several studies highlighted the often neglected maternal impact of FGR. A multicentre trial by Feenstra et al. revealed that maternal vascular malperfusion contributes to FGR in late pregnancy. Maternal vascularization at the feto-maternal unit in humans and mice involves uterine vasodilation and vessel remodeling upon trophoblast invasion with the help of immune cells. Using a specific deletion of the progesterone receptor in mouse dendritic cell, Thiele et al. showed that impairment of the cross talk between progesterone and dendritic cells leads to a reduction of pregnancy-protective immune cells leading to a poor placentation and FGR. These findings are corroborated by studies on pregnant women from Dunk et al., where defective progesterone-mediated poor decidualization is associated with an elevated mature dendritic profile and the failure of utero-vascular remodeling in FGR.

This review opens discussion about functional connections between placental malformation/malfunction and cardiovascular as well as brain diseases in adults. At this point it needs to be mentioned that not only FGR infants could develop a cardiovascular disease later in life, but there are long term cardiovascular consequences for mothers who develop preeclampsia during pregnancy, a topic which is very interesting however, not highlighted in our collection.

Langmia et al. summarize the effects of prolonged adverse maternal environment such as diabetic pregnancies as well as undernutrition and hypoxic conditions on placental genetic/epigenetic modifications, which in turn contribute to FGR combined with cardiovascular programming in rodents and humans. Malhotra et al. turn attention to consequences of growth restriction for the neonate after birth, and brings together a review of literature to show that FGR is associated with respiratory, cardiovascular, and neurological morbidities after birth that may require extra management in neonatal intensive care. The presence and severity of neonatal and longer term respiratory, cardiovascular, and neurological deficits reflect whether FGR is early- or late-onset, the severity of growth restriction, the degree of fetal cardiovascular adaptation, and the gestation at birth. Neonatal pathology is substantiated by Villamor-Martinez et al. who show that FGR but not SGA babies maintain a patent ductus arteriosus after birth which again emphasizes the importance to discriminate between SGA and FGR in clinical studies. The pulmonary consequences of FGR are examined by Allison et al. in FGR and appropriately-grown preterm lambs, together with the potential therapeutic effects of early intervention with umbilical cord blood stem cells.

Obstetric management can improve catch-up growth of FGR babies and also neurodevelopmental and behavioral outcomes (van Wyk et al.). When growth restriction is evoked by calorie restricted diet during rat pregnancy, intervention with lactoferrin ameliorates FGR, and has a neuroprotective effect as shown by van de Looij et al. Clinical investigations for health consequences of the offspring need to carefully discriminate between babies only with FGR, with FGR and premature birth, and babies defined as SGA babies. In this context Morsing et al. could demonstrate that reduced brain volumes as determined by MRI, normally judged as a consequence for FGR, are more likely due to premature birth.

Many studies deal with the consequences of an adverse maternal environment for brain development in growth restricted offspring. A good deal of those maternal insults summarized by Baud and Berkane include dysregulation of steroid hormones and growth factors that mediate placental feto-maternal exchange for fetal growth and neurodevelopment. The longer term neurological consequences of placental insufficiency and FGR are increasingly well-understood, particularly as standardized definitions of FGR and SGA are used, together with standardized assessment tools (Vollmer and Edmonds). Hartkopf et al. examined the origins of altered neurodevelopmental function in FGR offspring, and observed that fetal responses show early evidence of altered brain development, while at 2 years of age, children born FGR were found to have neurodevelopmental delays. Indeed the review by Vollmer and Edmonds describes that school-age children who were born growth restricted are more likely to demonstrate cognitive, behavioral, and/or attention deficits and this is particularly so when FGR and preterm birth are co-morbidities.

The review of Fleiss et al. summarizes research and clinical efforts to identify known pathogenic mechanisms that contribute to FGR and, in particular, neurodevelopmental compromise, and highlight knowledge gaps that require addressing. Quite sensibly, they demand a more concerted research focus and global networking on this complex topic. Yawno et al. examined the ontogeny of cerebellar (mal-)development in the final trimester of fetal sheep FGR and showed that altered brain cerebellar growth is evident antenatally, and continued *in utero* compromise leads to progressive worsening in development of the cerebellum. Together, these preclinical and clinical studies provide good evidence that altered brain development begins *in utero* due to placental compromise, and therefore early diagnosis and early intervention to improve neurological function in FGR offspring are key research areas.

## Conclusion

The collection of articles presented here in this special issue aimed to describe the various causes and many of the consequences for a baby developed in an adverse intrauterine environment wherein placental insufficiency leads to fetal growth restriction. We described the maternal insults or pregnancy compromise that may seriously affect fetal growth and well-being of the offspring including pregnancy diseases, eating disorders, infection/inflammation, and hormonal imbalance. It is however apparent that fetal growth restriction is primarily caused by placental dysfunction, leading to chronic fetal hypoxia and hypoglycaemia. It is these consequences of placental insufficiency that lead to reduced fetal growth overall, and suboptimal organ development. FGR is associated with lifelong burden of chronic diseases including metabolic, respiratory, cardiovascular and neurological deficits that greatly impact on individuals and society. At this time it remains that adequate diagnostic approaches as well as therapeutic interventions are lacking. The many studies in animals as well as in clinical trials reveal how complex and difficult it is to define a clear phenotype, and as a consequence, to implement an appropriate management strategy during pregnancy, a period which has high ethical limitations in treatment. Thus, in this topic we have described that FGR is strongly linked to altered neonatal and long-term outcomes, and research to improve outcomes must be based on an improved understanding of the pathophysiological mechanisms that alter organ development and growth. Herein our author contributors propose better collaborative research networks that will continue working toward standardized diagnosis and outcome assessments, and will continue research to increase our understanding of the complex changes that occur due to placental insufficiency, so that improved clinical management and treatment is implemented to reduce the burden of FGR.

## Author Contributions

All authors contributed as editors of this Research Topic, and wrote and edited this editorial.

### Conflict of Interest

The authors declare that the research was conducted in the absence of any commercial or financial relationships that could be construed as a potential conflict of interest.

